# Secondary torsion of vermiform appendix

**DOI:** 10.4103/0974-2700.62126

**Published:** 2010

**Authors:** Imtiaz Wani, Muddasir Maqbool, Tariq Sheikh

**Affiliations:** Department of General Surgery, SMHS Hospital, Srinagar, Kashmir, India

Sir,

We report a case of secondary torsion of vermiform appendix in an adult female. Torsion of the right ovary, which had pericystic adhesions to the vermiform appendix, had led to its simultaneous torsion. Histopathology of the ovary documented mucinous cystadenoma of ovary and there was a markedly congested appendix.

**Figure 1:**
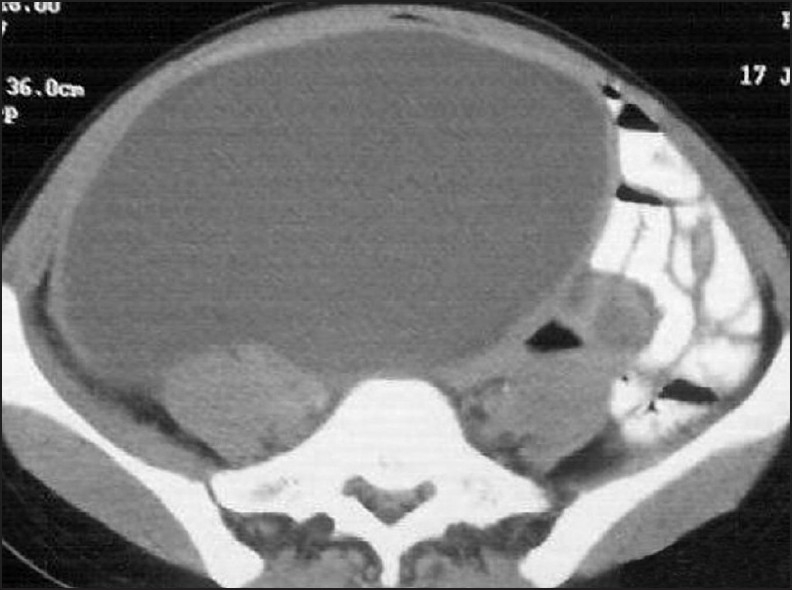
Computed tomography scan showing large cyst in the abdomen arising from the right adenexa

**Figure 2:**
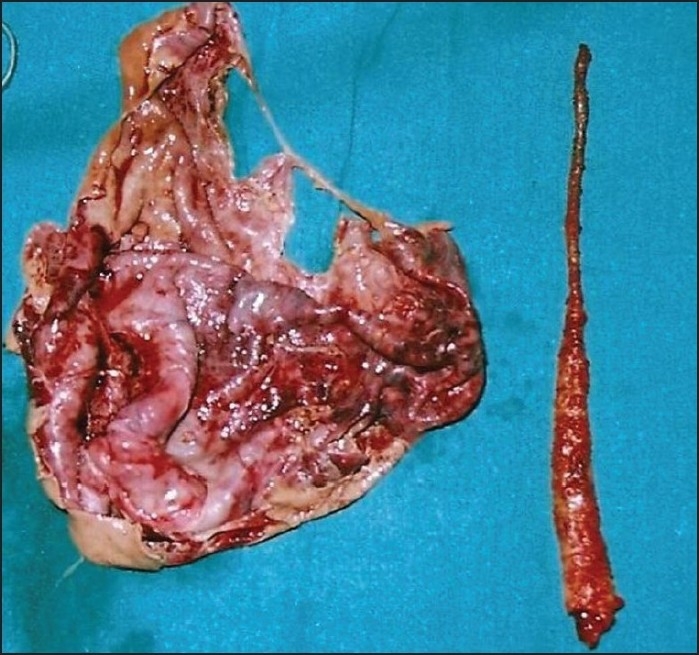
Right ovary and appendix

A 38-year-old female presented with 6 days’ history of right lower abdominal pain and fever. For the last 2 days she had had associated nausea, vomiting, and anorexia. The patient had a large right lower abdominal swelling which she had been aware of for the last 7 months, but she had not disclosed this to anyone. Per abdominal examination, showed a tender, globular, firm swelling measuring 19×38×7 cm in size. A computed tomography (CT) scan revealed a large cyst arising from the right adenexa and measuring 21×42×12 cm [Figure 1]. On exploratory laparotomy a large cyst was seen arising from the right ovary. There was a clockwise torsion of 180° of this cyst-bearing ovary. On the posterior aspect of the torsed ovary, a free-lying (pelvic) vermiform appendix was adherent; it was twisted at its distal end and a mucous collection was seen in the proximal part. A right salpingoophorectomy was done, along with appendectomy [Figure 2].

**Figure 3:**
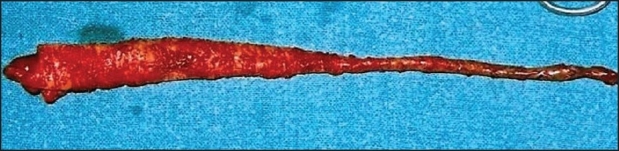
Twisted appendix with the point of twist being in the distal part

The appendix was about 20 cm in length with the point of twist about 7 cm from the base. A clockwise rotation of 180° was noted [Figure 3]. Histopathology of the ovary confirmed benign mucinous cystadenoma. The appendix showed congestion and mild inflammatory infiltrate. The postoperative period was uneventful and the patient was discharged with appropriate advice.

Torsion of the appendix is rare. The clinical presentation is indistinguishable from acute appendicitis.[1] Numerous causes have been cited in literature for torsion of appendix. Secondary torsion is associated with fecalith, lipoma, intussusception, malformation of appendix, and cystadenoma of appendix; adhesions may also rarely lead to torsion of the appendix.[2] Torsion of the appendix can mimic ovarian torsion.[3] Mucinous cystadenoma comprises 20% of all benign tumors and is the second most common benign epithelial neoplasm of the ovary after serous ovarian adenoma.

Torsion of appendix is rarely diagnosed preoperatively. Adhesions can lead to a twist in the distal part of the appendix. Appendectomy is the treatment of choice for torsion of appendix
